# Mr. Proctor, on the Unguentum Hydrargyri

**Published:** 1799-06

**Authors:** John Proctor


### *To the Editors of the Medical and Physical Journal.*

Gentlemen,

If the following communication accords with the plan of "*The Medical and Physical Journal,*" and you think it worth inserting, your notice of it will much oblige

Yours,

John Proctor, Jun.

The *unguentum hydrargyri* being a medicine much used, and the preparation of it rather troublesome, several expedients have been tried to facilitate the process; most of which are, however, more or less exceptionable, either on account of injuring the colour of the ointment, giving it a bad smell, or of being productive of disagreeable effects in the using of it: such such are the ol. sulphurat. the turpentines, &c. An improvement is noticed in No. 6, of the "*Phil. Mag.*" which is, the addition of a very minute portion of flor. sulph. (sulph. sublimat.) which I find, upon trial, extinguishes the quicksilver very readily, but the ointment was of a bad colour; and a larger quantity of sulphur would probably make it almost black. I was led to try a little empyreumatic or rancid fat, from the hint given in the following note in "*Fourcroy's Chemistry,*" vol. ii. p. 248, first edition, and found it answer beyond any thing I had ever used before for this purpose. It not only combines in a very short time with the quicksilver, but the colour of the ointment is equal, if not superior, to any that can be made with the purest fat without this addition. "In this ointment, the particles of the mercury do not merely seem to be distributed and interspersed among the particles of the fat, without any adherence or chemical union: on the contrary, the oily matter of mercurial ointment very quickly becomes rancid; and we know that rancidity, or incipient acidification, is always the consequence of the combination of oil with some other substance (now known to be oxygen, which it seems disposed to absorb more quickly by being united with a metallic substance). When the ointment is old, if we rub a portion of it between two bits of paper, the whole of the oil is absorbed, without leaving any globules of mercury visible behind it: but when we treat mercurial ointment, recently prepared, in the same manner, we can very readily perceive a great number of metallic particles quite distinct. M. Beaumé took equal quantities of mercurial ointment; one of which was newly made, and the other become slightly rancid by keeping. He kept both of them in a state of liquifaction during eight days, in a degree of heat much below what could possibly decompose the fat. The newly-made ointment allowed three drachms of mercury to separate; the other, which was rancid, only one drachm and a half. All these observations do not allow us to doubt of the reality of the combination; they pointedly prove, that what we call the extinction of mercury in fat, is not purely the effect of mechanical division, since those two substances exert a slow spontaneous action upon one another, from which a more intimate union at length results. This is much confirmed, by observing the difference in colour and consistence between old and new ointment. New made ointment is of a very light colour, and extremely soft; while what has been kept for some time is much darker in colour, and much firmer in consistence: a sufficient proof of some change in the intimacy of their union. We are in the next place to enquire in what state the mercury unites with the fat, whether in form of a metal, or in form of a calx (oxyd).

"When old mercurial ointment is converted into a saponaceous compound by the addition of caustic alkali, there is always a quantity of fluid mercury mercury feparated from the mixture, the fat forfaking the mercury to unite with the alkali. Mercurial ointment is alfo decompofed by the action of ether upon it. When a fmall quantity of good mercurial ointment is put into a flafk, which is two thirds full of ether and diftilled water, and the mixture frequently fhaken, the mercury foon begins to precipitate, carrying a fmall portion of fat along with it, which gives the mercury the appearance of a calx; but this fat foon difappears, and the mercury unites in the form of metallic globules, by fimply drying it upon bibulous paper. By this analyfis, we collect almoft the whole of the mercury in a fluid ftate. In reviewing all thefe facts carefully, it feems probable, that the mode in which mercury combines with fat, more refembles the amalgamation of the metals with mercury, than their diffolution in acids, as the mercury is taken up in a metallic ftate, and not calcined (oxydated); the fatty matter ferving the purpofe of a folvent to the mercury in the preparation of mercurial ointment, in the fame way that mercury itfelf ferves the purpofe of a folvent to the other metals, in the combination of the different amalgams."

I have copied the whole of this note, for the purpofe of introducing fome remarks upon it, which may perhaps explain more clearly the nature of this combination. I confider mercury to be fimply mechanically divided by being triturated (till totally extinguifhed) in *pure fweet* fat; for upon rubbing the mixture upon the furface of any body which will either abforb the fat, or allow it fpace to be diffufed upon, the mercury prefently re-unites into fmall globules, as in its original ftate; and this happens from the connection between it and the dividing matter (fat) being deftroyed. But when the ointment has been kept fome time, no fuch effect takes place, becaufe the union becomes more intimate in confequence of the fat undergoing a change, by which it really does diffolve part, and in time, the whole of the mercury; and this change is no other than a gradual acidification, which it feems more readily to undergo from its combination with the mercury, by the abforption of oxygen from the atmofphere. The fmell, which would betray its rancidity, or acidification, cannot be perceived till all the mercury is combined with the febacic acid, when the fuperabundant quantity of fat, having no metal to neutralize, or rather to faturate its acid, will of courfe become fenfible to the fmell. As a proof of this, old mercurial ointment will readily take a confiderable addition of frefh mercury, and in this way I at once prepare the ung. hydrarg. fort. by adding a proportionable quantity of mercury to a weaker ointment, confifting of one part of the metal to four of fat.

Led by this confideration of the nature of mercurial ointment, I found, upon trial, that a fmall portion of rancid fat extinguifhed a large one of quickfilver, quickſilver, without communicating any diſagreeable ſmell to the ointment when made. Should the fat be any way ſtiff or hard, it may be ſoftened with a little oil of the ſame nature. Train-oil anſwers very well; as it ſpontaneouſly melts from the blubber, before it has undergone any change from heat, it is quite ſweet, and free from ſmell: perhaps the large quantity of animal mucilage that is combined with it, may haſten its change in the ordinary temperature of our climate in ſummer; but, as in the caſe of other animal fats, its ſmell is quite deſtroyed, when combined with a metal. The cauſe of the rancidity of oil, may furniſh ſome hints for their edulcoration; but, beſides neutralizing and waſting their diſengaged acid, it is neceſſary to ſeparate the mucilage which is combined with them, either by preſſure, or in the proceſs of obtaining them by boiling, and which is depoſited from ſeveral in conſiderable quantity, on ſtanding at reſt for ſome time. What is the preciſe difference between the mucilage of animal and vegetable oils; and in what degree do they contribute to accelerate the rancidity or putreſcency of theſe oils?—The firſt of theſe ſtates I conſider to be owing to the abſorption of oxygen, and the other to the diſengagement of volatile alkali.

On the principles of the foregoing theory, I have tried with equal ſucceſs, the effects of rancid vegetable oil, in making the emplaſt. litharg. and have found the proceſs much ſhortened when I have uſed this kind of oil, and the plaiſter, when made, equally good, if not better, than that made with ſweet oil. Upon the whole, I conſider the mercurial ointment to be a true ſebat of mercury, combined with a more or leſs conſiderable portion of *uncombined* fat, according to the length of time it has been kept. The experiment with ether is nearly ſimilar to the mixture of any other metal diſſolved in an acid with this liquor. The ſolution of gold in aqua regia, and the tinct. ferri. mur. when mixed with ether, exhibit nearly the ſame phenomenon, viz. the reduction of the oxyd, by the abſtraction of its oxygen.

It is far from being conſiſtent with a true knowledge of chemiſtry, to compare the combination of mercury with fat to an amalgam; there is not the moſt diſtant analogy between theſe two compounds. Were I to draw a compariſon between a mixture of mercury with other metals, and any other mixture reſembling it, I would beg leave to mention that of wax, reſin, and oil, which agrees exactly with it, as to its ſtate, though it differs materially from it in the nature of its component parts. Mercury is in the one compound exactly what the oil is in the other; with reſpect to ſolidity, they differ in nothing from the other ingredients of their reſpective combinations, but in being fluid at a lower temperature, or containing a greater quantity of of latent heat, a portion of which they yield to the other parts of the compofition, and fo form a mafs of an homogeneous confiftence and appearance. The fimilarity is rendered ftill more conformable, if we fuddenly cool a mixture of the unctuous fubftances, when each of them will be diftinctly perceptible, by the difference of confiftence.

Other chemical fubftances are capable of producing their effects in the fyftem by abforption, when externally applied, as well as mercury: arfenic and tartarized antimony have furnifhed feveral inftances of this mode of action.

