# Errata in Vol. I

**Published:** 1799-08

**Authors:** 


### ERRATA IN VOL. I.

No. iv. p. 359, l. 9, for "oil," read, "*fixed oils.*"

No. iv. p. 359, l. 10, for "wasting," read, "*washing off.*"

No. iv. p. 359, l. 24, for "uncombined," read, "*unaltered*"

